# Enhancing the nutritional value of sweet pepper through sustainable fertilization management

**DOI:** 10.3389/fnut.2023.1264999

**Published:** 2023-11-29

**Authors:** Vasile Stoleru, Ionel Mangalagiu, Dorina Amăriucăi-Mantu, Gabriel-Ciprian Teliban, Alexandru Cojocaru, Oana-Raluca Rusu, Marian Burducea, Gabriela Mihalache, Mihaela Rosca, Gianluca Caruso, Agnieszka Sekara, Gerard Jităreanu

**Affiliations:** ^1^Department of Horticulture, “Ion Ionescu de la Brad” University of Life Sciences, Iași, Romania; ^2^Department of Biochemistry, “Alexandru Ioan Cuza” University of Iasi, Iași, Romania; ^3^Department of Public Health, “Ion Ionescu de la Brad” University of Life Sciences, Iași, Romania; ^4^Research and Development Station for Aquaculture and Aquatic Ecology, Alexandru Ioan Cuza University, Iași, Romania; ^5^Integrated Center of Environmental Science Studies in the North Eastern Region (CERNESIM) “Alexandru Ioan Cuza” University, Iași, Romania; ^6^Department of Agricultural Sciences, University of Naples Federico II, Naples, Italy; ^7^Department of Horticulture, Faculty of Biotechnology and Horticulture, University of Agriculture, Krakow, Poland; ^8^Department of Plant Sciences, “Ion Ionescu de la Brad” University of Life Sciences, Iași, Romania

**Keywords:** *Capsicum annuum* L., cultivars, fertilization regimes, minerals, phytochemicals, proximate composition, yield

## Abstract

**Introduction:**

The need for healthy foods has become a major concern in our modern world, as the global population continues to grow and environmental challenges intensify. In response to these challenges, researchers have started to explore a range of sustainable solutions, including organic farming practices, precision agriculture, and the development and testing of innovative biofertilizers. Consistent with these ideas come the aim of this study, which sets out to give new insights into the cultivation of two sweet pepper cultivars with economic and nutritional importance in Romania.

**Methods:**

Two sweet pepper cultivars (Blancina and Brillant), chemically (Nutrifine^®^), organically (Orgevit^®^) and biologically (Micoseed^®^) fertilized were cultivated over the course of two years (2019 and 2020), between April and October, in high-tunnel, by following a split-plot design with three replications. Production parameters (number of fruits, fruit weight, yield), proximate composition (water content, dry matter, total soluble solids, acidity, ash), the content of phytonutrients (polyphenols, lycopene, β-carotene, antioxidant activity), phytochemical composition (phenolic compounds) and minerals (macro- and micro-elements) were analyzed in order to determine the impact of fertilization on the quality of sweet peppers.

**Results:**

The results showed that the biological and organic fertilizations had a significant positive impact on most of the parameters analyzed, starting with yield and continuing with acidity, phytonutrient content (total phenolic content, lycopene, β-carotene), antioxidant activity and phytochemical composition (chlorogenic acid, *p*-coumaric acid, quercetin and isoquercetin). Only in the case of mineral content, the chemical treatment gave better results compared with the organic and biological fertilizers.

**Conclusion:**

Overall, this study provides valuable information on the potential of organic and biological fertilizers to enhance the nutritional value of sweet peppers from Blancina F1 and Brillant F1 cultivars, paving the way for subsequent research aimed at achieving superior quality and increased yields.

## 1 Introduction

Sweet pepper (*Capsicum annuum* L.) is a widely cultivated vegetable crop belonging to the *Solanaceae* family, which also includes tomato, potato and eggplant. These species are typically grown as annuals and can be cultivated in a wide range of climates and soil types. Sweet pepper is a warm-season crop that is grown in many parts of the world, and it is valued for its culinary and nutritional qualities. There are numerous varieties of sweet peppers, with different colors, shapes, sizes, and flavors (1). In accordance with FAO, in 2021 the global production of chillies and peppers (*Capsicum* spp. and *Pimenta* spp.) was 36,286,643.77 tons (2). Sweet peppers are rich in vitamins A and C, potassium, folate, fiber as well as other minerals and antioxidants. They also contain bioactive compounds that have been associated with various health benefits, such as reducing the risk of cancer, diabetes, and cardiovascular diseases (3). The quality of sweet peppers is an important factor that affects their market value and consumer acceptance. Pepper quality is a multi-faceted concept that encompasses various factors, such as appearance, flavor, aroma, and pungency. When it comes to pungency, the presence of capsaicinoids plays a significant role. Quality can be influenced by various factors, including genetics, environment, and cultural practices (4). The nutritional value of sweet peppers is an important aspect of quality, as it affects their health benefits and marketability. Nutritional values of sweet pepper depend on their cultivar., degree of ripeness, and growing conditions (5, 6). In addition to their nutritional value, peppers also contain carotenoids, flavonoids, and phenolic acids, that have been shown to have various biological activities, such as antioxidant, anti-inflammatory, and anticancer properties (3, 7). As in the case of nutritional value, the levels of these compounds can vary among cultivars and can be influenced by environmental factors and cultivation practices such as fertilization (8, 9). Fertilization is a critical component of modern agricultural practices, and the use of different types of fertilizers can significantly affect the production and quality of sweet peppers. Fertilization can be categorized into chemical, biological, and organic types. Chemical fertilizers are widely used in modern agriculture, but they can have negative environmental impacts and can also affect the quality of crops. For instance, in a study done by Hallmann et al. (10), the content of dry matter, total flavonoids and phenolic acids were significant lower in chemically fertilized peppers than in organic fertilized peppers; Selvakumar et al. (11), registered lower amounts of macronutrients (N, K) in the peppers treated with chemical fertilizer compared with organic fertilizer or Ashour et al. (12), recorded in hot peppers a better ascorbic acid content, antioxidant capacity, flavonoids or phenols due to the application of organic fertilizers. Regarding the negative impact on environment, chemical fertilization is responsible for soil acidification, emission of greenhouse gases, accumulation of mineral salts, imbalances in the soil microbiota, eutrophication of water bodies (13). Therefore, in recent years, there has been growing interest in the use of biological and organic fertilizers, which can improve soil fertility and promote sustainable agriculture (14). Green manure, chicken manure, farm manure, earthworms, seaweeds (organic fertilizers) or microorganisms (biological fertilizers) have been shown to positively affect the production and accumulation of bioactive compounds in vegetables, including sweet peppers (3, 10, 12, 15–18). These fertilizers provide nutrients to plants through the natural decomposition of organic matter, which promotes the growth of beneficial microorganisms in the soil. The microorganisms in turn enhance the availability of nutrients to the plants, improve soil structure and water-holding capacity, and stimulate root growth and plant metabolism (19). This increased plant growth and metabolism can lead to higher levels of bioactive compounds, such as carotenoids, flavonoids, and phenolic acids, in the fruits. In addition, some organic and biological fertilizers can also stimulate the production of plant hormones and enzymes, which can further enhance the accumulation of bioactive compounds in the fruits (20, 21). The organic food and superfoods market has been on the rise due to growing consumer interest in healthier and more sustainable food options. It was valued at USD 188.35 billion in 2021 and is expected to experience a Compound Annual Growth Rate of 13.0% from 2022 to 2030 (22). This growth is driven by the health benefits associated with organic products and changes in consumer buying habits.

Given that ensuring the productivity and quality of vegetable crops is of great importance to satisfy the increasing needs of the expanding world population, the main objective of this study was to investigate the impact of three types of fertilizers (chemical, biological, and organic) on the yield, nutritional value and bioactive compound content of two sweet pepper cultivars (Blancina F1 and Brillant F1), for which limited information are available. Fertilizers, whether organic, chemical or biological as a result of their mineral composition support plant growth differently, as shown for example by the results of the study conducted by Muscolo et al. (23), which outlines the effects of three organic fertilizers on phytochemicals and antioxidant properties of red sweet pepper cv. Topepo. This study revealed that the highest levels were detected in fruits of red Topepo treated with compost from vegetable wastes, values significantly higher compared to those measured in the fruit of peppers treated with compost from olive wastes and horse dung. Therefore, choosing the adequate fertilizer contributes to the achievement of the greatest crop yield and crop quality. So, by evaluating the relationship between fertilization with Nutrifine^®^, Orgevit^®^ and Micoseed^®^ and fruit quality in sweet pepper cultivars, this study aims to provide valuable information for the development of sustainable agricultural practices in the cultivation of these sweet peppers with great economic and food importance in Romania. Furthermore, the results of this research will contribute to a better understanding of the role of fertilization in the production of high-quality sweet pepper.

## 2 Materials and methods

### 2.1 Experimental design

**Figure 1:**
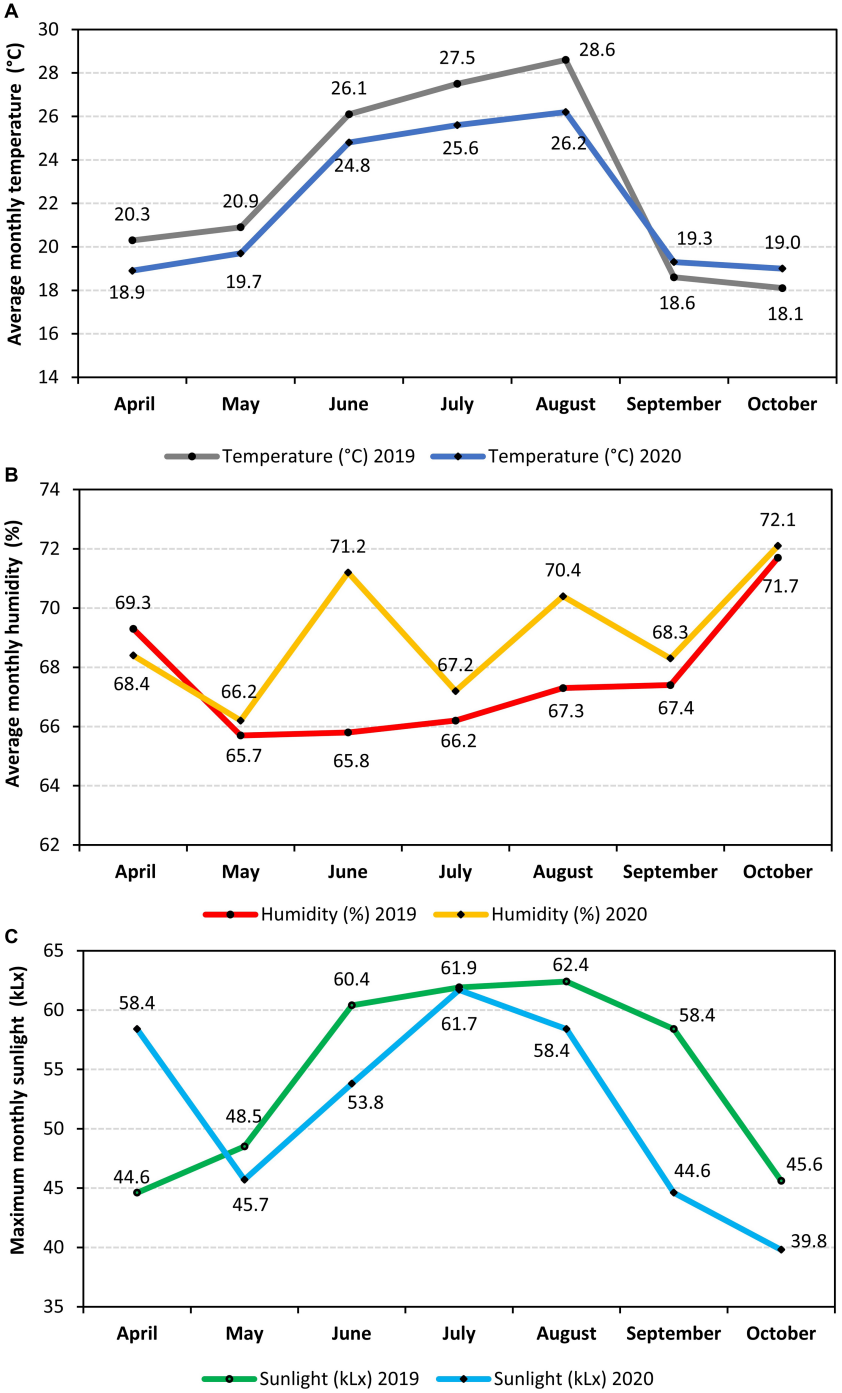
Climatic conditions during the experiment (2019–2020): **(A)** – average monthly temperature; **(B)** average monthly humidity; and **(C)** – maximum monthly sunlight.

A two-year (2019 and 2020) high-tunnel experiment was carried out at “V. Adamachi” Research Farm (*N* = 47°11′76″ *E* = 27°33′71″) of Iasi University of Life Sciences to investigate the combined effects of three types of fertilizations and two sweet pepper genotypes (*Capsicum annuum* L.) on both production and nutritional value. The experimental design was employed with two pepper cultivars (first factor) and three types of fertilization (second factor), along with an untreated control. The experiment followed a split-plot arrangement with three replicates, with each plot containing 18 plants, covering a total area of 7.2 square meters. The plants were spaced at 100 × 40 cm intervals, resulting in a density of 2.5 plants per square meter. The soil type in the experimental area was identified as loam-clay chernozem, with the following characteristics: pH 7.20, electrical conductivity of 478 μS·cm^−2^, CaCO_3_ content of 0.41%, organic matter content of 28.56 mg·kg^−1^, C/N ratio of 5.91, nitrogen content of 2.8 g·kg^−1^, and phosphorus content of 32 mg·kg^−1^ (24, 25). Detailed information regarding the climatic conditions throughout the experimental period is depicted in Figure 1.

### 2.2 Sweet pepper cultivars and the types of fertilization used

Two sweet pepper cultivars were used, Blancina and Brillant, which are recognized for their high yield, fruit quality (26, 27), and resistance to pests such as tomato spotted wilt virus (27). The seeds of ‘Blancina’ sweet pepper were produced by Enza Zaden, Netherlands and Brillant seeds by ZKI, Hungary. The experimental crop was established in mid of April by transplanting the 8-weeks-old seedlings and ended in October.

In this study, three types of fertilization were tested: chemical (Nutrifine^®^), organic (Orgevit^®^), and biological (Micoseed^®^). Each fertilization type represented a different approach to provide the optimum amount of nutrients to the plants.

Chemical fertilization was applied in three doses: the first one of 200 kg·ha^−1^ NPK (20-20-20) was applied prior to transplanting, followed by two applications during the growth period of 300 kg·ha^−1^ NPK (9–18-27 + 2MgO).

Organic fertilization was applied in two doses: the first one of 1,250 kg·ha^−1^ was applied before transplanting and the second one of 750 kg·ha^−1^ was applied 30 days after planting. Orgevit^®^ is a chicken manure granular fertilizer with 65% organic matter (OM) and contains nutrients such as nitrogen (N), phosphorus (P_2_O_5_), potassium (K_2_O), magnesium (MgO), iron (Fe), manganese (Mn), boron (B), zinc (Zn), copper (Cu), and molybdenum (Mo).

Biological fertilization was applied in two equal doses of 15 kg·ha^−1^: the first one just before transplanting and the second one 30 days after planting. Additionally, 5 L·ha^−1^ of Nutryaction^®^ was integrated according to the manufacturer’s recommendations. Micoseed^®^ is a biological product containing arbuscular mycorrhizal fungi spores of *Claroideoglomus etunicatum*, *Funneliformis mosseae*, *Glomus aggregatum*, and *Rhizophagus intraradices*, while Nutryaction^®^ contains various beneficial fungi and bacteria, such as *Trichoderma*, *Streptomyces*, *Bacillus*, and *Pseudomonas*.

Throughout the growing season, standard agricultural practices were implemented, including drip irrigation, stringing, and the removal of branches and old leaves. Pest and disease management were carried out using products that complied with organic regulations, ensuring the sustainability and health of the crop.

### 2.3 Production indicators of sweet peppers

The production indicators were the number of fruits, fruit weight, and yield. The formula for yield calculation was: yield (kg·ha^−1^) = (plants/ha × fruits/plant × average fruit weight)/1,000 (28).

### 2.4 Proximate analysis of sweet peppers

Proximate analyses of fruits included: water content, dry matter, total soluble solids, acidity, and ash.

For the water and dry matter content the samples were placed in a stove (Sanyo MOV-112F) at 70°C until a constant weight was achieved. The water content was calculated by subtracting the weight of the dried sample from the initial weight, and then dividing it by the initial weight. The dry matter content was calculated by subtracting the water content from 100%.

Total soluble solids (TSS) of peppers juice were measured with a digital refractometer (RX5000α, Atago, Tokyo, Japan).

The acidity was determined by titration using a titrator (808 Titrando, Metrohm, Wesbury, NY, United States) on 6.0 g of peppers juice diluted with 60 mL of ultra-clean water and results were expressed in citric acid content (29).

The ash content was determined following the AOAC method (30).

### 2.5 Analysis of total content of phytonutrients

Phytonutrient analyses involved quantifying total polyphenols, lycopene, β-carotene, and antioxidant activity in the sweet pepper extracts.

The extracts used for determination of polyphenols and individual phenolic compounds were prepared from commercially mature fruits (BBCH 703-705). The fruits were dried using a stove (Sanyo MOV-112F) at 70°C until constant weight, then ground into small fragments (0.1–1 mm). Aqueous extraction was performed at 38°C for 1 h, then samples were filtered and frozen at −24°C for further analysis.

The total polyphenols content was determined using the Folin-Ciocâlteu method as described by Liu et al. (31). A mixture of 2 mL of the extract, 10 mL of Folin-Ciocâlteu reagent (diluted 1:10), and 8 mL of a 75% Na_2_CO_3_ solution was prepared and allowed to react for 120 min at room temperature. After the reaction, the mixture absorbance was measured at 750 nm using a Jasco V530 spectrophotometer. The absorbance values were then compared to a gallic acid calibration curve to determine the polyphenols content. The results were expressed in micrograms of gallic acid equivalent (GAE) per milliliter (μg·100 mL^−1^).

For lycopene and β-carotene, the extracts for analysis were prepared using fresh biomass. The lycopene content was determined using the spectrophotometric method described by Davis et al. (32). Spectrophotometric readings were performed at 472 nm.

For the determination of β-carotene, the method described by Cadoni et al. (33) was used, and spectrophotometric readings were performed at 452 nm.

Antioxidant activity was measured according to Musa et al. (34). Approximately 10 g of finely ground plant material was extracted in 100 mL of 80% aqueous ethanol solution at room temperature for 1 h. The extracts were then filtered. Extracts (0.05 mL) were mixed with 2.95 mL of DPPH (2,2-Diphenyl-1-picrylhydrazyl) solution at three different concentrations (10, 5, and 2.5 mg/mL). The absorption was measured at 420 nm after a 5-min reaction time using methanol as a blank and the percentage of free-radical scavenging activity was calculated. The Trolox (6-hydroxy-2,5,7,8-tetramethylchroman-2-carboxylic acid) equivalent antioxidant capacity (TEAC) was calculated by comparing the percentage of free-radical scavenging activity of the extracts with the Trolox standard curve.

### 2.6 Analysis of phytochemical composition of sweet pepper fruits

Phytochemical composition was determined by the analyzing the phenolic compounds using RP-HPLC with a UV detector according to Vlase et al. (35). A total of 18 standard compounds including caffeic acid, chlorogenic acid, p-coumaric acid, ferulic acid, gentisic acid, sinapic acid, caftaric acid, kaempferol, apigenin, rutin, quercetin, quercetin-3-β-D-glucoside, hyperoside, myricetin, isoquercetin, fisetin, patuletin, and luteolin were used for the analysis. The HPLC system used was an Agilent 1,100 Series with a reversed-phase column (Zorbax SB-C18, 100 mm × 3.0 mm, 3.5 μm) operating at a temperature of 48°C. The mobile phase consisted of methanol and 0.1% acetic acid, with a flow rate of 1 mL/min and an injection volume of 5 μL. The elution gradient started at 5% methanol and increased to 42% methanol over 35 min, followed by an isocratic elution with 42% methanol for 3 min. UV detection was per-formed at 330 nm for the first 17.5 min and then at 370 nm for the remaining time. Quantitative determination was carried out using an external standard method, with a calibration curve that showed good linearity in the range of 0.5–50 μg/mL, and a regression coefficient (R^2^) of 0.9943.

### 2.7 Analysis of mineral content of sweet pepper

The determination of mineral concentration in the pepper was carried out using the method of atomic absorption spectrometry. First, the fruits were dried in an oven at 105°C for 48 h. Subsequently, samples weighing 0.5 and 1 g were subjected to digestion. The minerals were extracted using a mixture of nitric acid (HNO_3_) and hydrochloric acid (HCl) in a 1:1 ratio, following the procedure outlined by Fernández-Ruiz et al. (36). The resulting solutions were then analyzed using the Contra 300 atomic absorption spectrometer (Analytik Jena, Göettingen, Germany).

### 2.8 Statistical analysis of the data

The results are expressed as means ± standard deviation (SD) for the two years of the experiment. Prior to statistical analysis, the data were assessed for normality using the Shapiro–Wilk test. Subsequently, a two-way analysis of variance (ANOVA) was performed. Significant differences between treatments were determined using Duncan’s test with a confidence level of 95% (*p* ≤ 0.05). The statistical analysis was conducted using SPSS software version 21 (IBM Microsoft, New York, United States).

## 3 Results

### 3.1 Effects of fertilizer treatments on production indicators of sweet peppers

The production indicators (number of fruits, fruit weight, and yield) of the two sweet peppers cultivars (Blancina and Brillant) fertilized with three types of fertilizers (chemical, organic, and biological) are presented in Table 1. The statistical analysis of the number of fruits showed an increase of 20% and 29%, respectively, for Blancina and Brillant plants’ chemically fertilized, compared to the control group. Increases were also observed for the plants of both cultivars treated with organic and biological fertilizers, but no significant difference were recorded as compared to the non-fertilized plants. As for the weight of the fruits, increases were observed for all the types of fertilization, with the highest values recorded for biological fertilization followed by organic and chemical fertilization for both cultivars. Regarding the yield, for Blancina cultivar., the biological fertilization resulted in the highest increase compared to the control group (56%), followed by chemical fertilization (37%) and organic fertilization (28%). The same trend was observed for Brillant cultivar., with increases of 53% for biological fertilization, 45% for chemical fertilization, and 22% for organic fertilization. Overall, Blancina cultivar was more productive than Brillant cultivar.

**Table 1:** Production indicators of sweet peppers.

Treatment	No of fruits per plant	Mean weight per fruit (g)	Yield (t·ha^−1^)
Blancina × Nutrifine^®^	23.6 ± 2.3a	150 ± 15ab	101.14 ± 10.05ab
Blancina × Orgevit^®^	19.7 ± 2ab	168 ± 17ab	94.55 ± 9.39abc
Blancina × Micoseed^®^	22.9 ± 2.3ab	176 ± 17a	115.15 ± 11.44a
Blancina × Control	19.6 ± 2ab	131 ± 13ab	73.36 ± 7.29bcd
Brillant × Nutrifine^®^	20.9 ± 2.1ab	134 ± 13ab	80.01 ± 7.95bcd
Brillant × Orgevit^®^	17.0 ± 1.7ab	139 ± 14ab	67.51 ± 6.71 cd
Brillant × Micoseed^®^	20.4 ± 2ab	145 ± 15ab	84.51 ± 8.4bc
Brillant × Control	16.2 ± 1.6b	119 ± 12b	55.08 ± 5.47d

Within each column values associated with the same lower-case letters are not statistically different at *p* ≤ 0.05 according to Duncan’s test.

### 3.2 Effects of fertilizer treatments on proximate analysis of sweet peppers

The results of the proximate analysis of sweet pepper fruits [water content, dry matter, total soluble solids (TSS), acidity, and ash] are shown in Table 2. The interaction between the cultivar and the type of fertilizer did not result in significant changes in terms of water content, dry matter, TSS and ash content regardless the cultivar or the type of fertilization. Only in the case of acidity the content of citric acid varied between 0.19 g citric acid·100 g^−1^ f.w. for unfertilized Blancina cultivar and 0.36 g citric acid·100 g^−1^ f.w. for Brillant biologically fertilized pepper plants; significant differences being recorded between the pepper fruits of ‘Brillant’ cultivar treated with biological fertilizer and the rest of the treatments (Table 2).

**Table 2:** Proximate composition of sweet pepper fruits.

Treatment	Water content (%)	Dry matter (%)	TSS (^o^Brix)	Acidity (g citric acid·100 g^−1^ f.w.)	Ash (g·100 g^−1^ d.w.)
Blancina × Nutrifine^®^	94.50 ± 2.28a	5.50 ± 1.06a	5.06 ± 0.98a	0.22 ± 0.05b	4.00 ± 0.70a
Blancina × Orgevit^®^	93.77 ± 1.99a	6.23 ± 1.20a	6.11 ± 1.18a	0.24 ± 0.05b	4.86 ± 0.94a
Blancina × Micoseed^®^	93.13 ± 1.98a	6.87 ± 1.33a	6.32 ± 1.22a	0.27 ± 0.06b	5.45 ± 1.05a
Blancina × Control	94.11 ± 1.92a	5.89 ± 1.14a	5.87 ± 1.13a	0.19 ± 0.04b	4.49 ± 0.86a
Brillant × Nutrifine^®^	94.40 ± 2.16a	5.60 ± 1.08a	5.21 ± 1.01a	0.24 ± 0.05b	4.10 ± 0.79a
Brillant × Orgevit^®^	93.27 ± 2.18a	6.73 ± 1.30a	6.18 ± 1.19a	0.27 ± 0.06b	5.36 ± 1.04a
Brillant × Micoseed^®^	93.06 ± 1.98a	6.94 ± 1.34a	6.83 ± 1.32a	0.36 ± 0.07a	5.52 ± 1.07a
Brillant × Control	93.94 ± 2.34a	6.06 ± 1.17a	5.69 ± 1.10a	0.22 ± 0.05b	4.66 ± 0.90a

Within each column values associated with the same lower-case letters are not statistically different at *p* ≤ 0.05 according to Duncan’s test.

### 3.3 Effects of fertilizer treatments on antioxidant and phytonutrient content of sweet peppers

The total content of phytonutrients including polyphenols, lycopene, β-carotene, and antioxidant activity of the sweet pepper extracts are presented in Table 3. The statistical analysis of the results showed significant differences between the treatments, for all the measured parameters (*p* < 0.05), indicating that fertilization had a significant impact on the phytochemical composition and antioxidant activity of the peppers. Biological and organic fertilization contributed the most to the improvement of nutritional quality of peppers, in both cultivars, while chemical fertilization had a less pronounced effect. For instance, the highest total phenolic content was registered under the biological fertilization of Blancina and Brillant peppers, with increases of 76% and 14% respectively, as compared to the control. The highest content of lycopene and β-carotene was recorded for the organic fertilization, with increases of 77% and 72% for Blancina cultivar and 59 and 54% for Brillant cultivar. Regarding, the antioxidant activity of peppers, a significant positive effect was observed under the biological fertilization, with increases of 53% (Blancina) and 25% (Brillant) as compared to the peppers from unfertilized plants.

**Table 3:** Antioxidant and phytonutrient content of sweet peppers fruits.

Treatment	Total phenolic (μg·100 mL^−1^)	Lycopene (mg·100 g^−1^ f.w.)	β-Carotene (mg 100 g^−1^ f.w.)	Antioxidant Activity (mmol Trolox·100 g^−1^ d.w.)
Blancina × Nutrifine^®^	273.05 ± 52.73c	8.55 ± 1.65c	15.27 ± 2.95b	113.89 ± 22.00c
Blancina × Orgevit^®^	414.23 ± 80.00bc	16.32 ± 3.15a	26.32 ± 5.08a	137.94 ± 26.64bc
Blancina × Micoseed^®^	649.52 ± 125.44a	13.57 ± 2.62ab	20.56 ± 3.97ab	194.70 ± 37.61a
Blancina × Control	367.17 ± 70.92bc	9.17 ± 1.77c	15.28 ± 2.95b	126.59 ± 24.45bc
Brillant × Nutrifine^®^	384.82 ± 74.32bc	8.80 ± 1.70c	15.71 ± 3.03b	134.60 ± 26.00bc
Brillant × Orgevit^®^	531.88 ± 102.72ab	15.94 ± 3.08a	25.71 ± 4.97a	141.32 ± 27.29bc
Brillant × Micoseed^®^	602.47 ± 116.36a	14.27 ± 2.76ab	21.62 ± 4.18ab	173.35 ± 33.48ab
Brillant × Control	526.00 ± 101.59ab	10.00 ± 1.93bc	16.67 ± 3.22b	138.66 ± 26.78bc

Within each column values associated with the same lower-case letters are not statistically different at *p* ≤ 0.05 according to Duncan’s test.

### 3.4 Effects of fertilizer treatments on phytochemical composition of sweet peppers

The phytochemical composition of sweet peppers is shown in Table 4. The content of chlorogenic acid ranged from 1.60 to 6.10 μg·100 mL^−1^, p-coumaric acid from 1.90 to 5.70 μg·100 mL^−1^, quercetin from 59.8 to 396.30 μg·100 mL^−1^ and isoquercetin from 1.00 to 4.80 μg·100 mL^−1^. The rutin and ferulic acid in sweet peppers fruits were detected in trace levels. The analysis showed that of the three fertilizers used, the biological one has the greatest influence on the phytochemical composition of sweet peppers, the fruits of Brillant × Micoseed^®^ containing the highest levels. Statistically, the levels of chlorogenic acid, p-coumaric acid, quercetin, and isoquercetin in sweet peppers treated with biological, organic and chemical fertilizers are significantly higher compared to the levels in the control samples (*p* < 0.05), revealing a significant positive impact of the used fertilizers on secretion of these phytochemical components.

**Table 4:** Phytochemical composition of sweet peppers fruits.

Treatment	Chlorogenic acid (μg·100 mL^−1^)	*p*-coumaric acid (μg·100 mL^−1^)	Rutin (μg·100 mL^−1^)	Ferulic acid (μg·100 mL^−1^)	Quercetin (μg·100 mL^−1^)	Isoquercetin (μg·100 mL^−1^)
Blancina × Nutrifine^®^	3.60 ± 0.40bc	2.20 ± 0.24d	tr	tr	88.80 ± 9.90de	1.30 ± 0.14c
Blancina × Orgevit^®^	5.20 ± 0.58ab	4.00 ± 0.45bc	tr	tr	171.00 ± 19.07bc	2.98 ± 0.33b
Blancina × Micoseed^®^	5.80 ± 0.65a	4.90 ± 0.55ab	tr	tr	220.6 ± 24.60b	4.10 ± 0.46a
Blancina × Control	1.60 ± 0.18d	1.90 ± 0.21d	tr	tr	59.8 ± 6.67e	tr
Brillant × Nutrifine^®^	3.90 ± 0.43b	2.90 ± 0.32 cd	tr	tr	142.00 ± 15.83 cd	1.00 ± 0.11c
Brillant × Orgevit^®^	5.20 ± 0.58ab	4.30 ± 0.48b	tr	tr	185.00 ± 20.63bc	2.50 ± 0.28b
Brillant × Micoseed^®^	6.10 ± 0.68a	5.70 ± 0.64a	tr	tr	396.30 ± 44.19a	4.80 ± 0.54a
Brillant × Control	2.20 ± 0.24 cd	2.70 ± 0.30 cd	tr	tr	60.70 ± 6.77e	tr

Within each column values associated with the same lower-case letters are not statistically different at *p* ≤ 0.05 according to Duncan’s test; tr – trace below detection limit 0.5 μg∙mL^−1^.

### 3.5 Effects of fertilizer treatments on mineral content of sweet peppers

The mineral content of sweet peppers grown under different type of fertilizers is displayed in Table 5. Compared to the control, the fertilization of sweet peppers has led to an increase in the mineral content in their fruits, the highest levels being determinate in sweet peppers grown with Nutrifine (chemical fertilizer). Under the action of chemical fertilizer, the potassium (K), calcium (Ca), and phosphorus (P) contents in Blancina’s fruits increased with 28%, 54%, and 72%, while in the Brillant’s fruits the increases were with 43%, 32%, and 51% compared to the control. Statistically, at *p* < 0.05, only the P content in Blancina’s fruits fertilized with Nutrifine^®^ and Orgevit^®^ were significantly higher compared with the level detected in control, implying that these fertilizers had a significant impact on content of this mineral. However, no significant differences were induced by organic, biological and chemical fertilizers on K, Ca and Mg contents in pepper’s fruits.

**Table 5:** The content of macroelements in sweet peppers fruits.

Treatment	K (mg·100 g^−1^ f.w.)	Ca (mg·100 g^−1^ f.w.)	P (mg·100 g^−1^ f.w.)	Mg (mg·100 g^−1^ f.w.)
Blancina × Nutrifine^®^	236.54 ± 45.68a	16.52 ± 3.19ab	13.79 ± 2.66ab	10.65 ± 2.06a
Blancina × Orgevit^®^	214.70 ± 41.47a	16.22 ± 3.14ab	13.33 ± 2.58ab	13.47 ± 2.60a
Blancina × Micoseed^®^	209.12 ± 40.39a	15.12 ± 2.92ab	11.83 ± 2.28abc	13.04 ± 2.52a
Blancina × Control	183.66 ± 35.47a	10.72 ± 2.07b	7.99 ± 1.55c	10.57 ± 2.04a
Brillant × Nutrifine^®^	254.62 ± 49.18a	18.52 ± 3.58a	15.79 ± 3.05a	15.25 ± 2.94a
Brillant × Orgevit^®^	222.14 ± 42.90a	16.42 ± 3.17ab	13.69 ± 2.64ab	14.77 ± 2.85a
Brillant × Micoseed^®^	209.96 ± 40.55a	16.08 ± 3.11ab	12.27 ± 2.37abc	12.99 ± 2.51a
Brillant × Control	178.00 ± 34.38a	13.97 ± 2.69ab	10.39 ± 2.01bc	11.13 ± 2.15a

Within each column values associated with the same lower-case letters are not statistically different at *p* ≤ 0.05 according to Duncan’s test.

**Figure 2:**
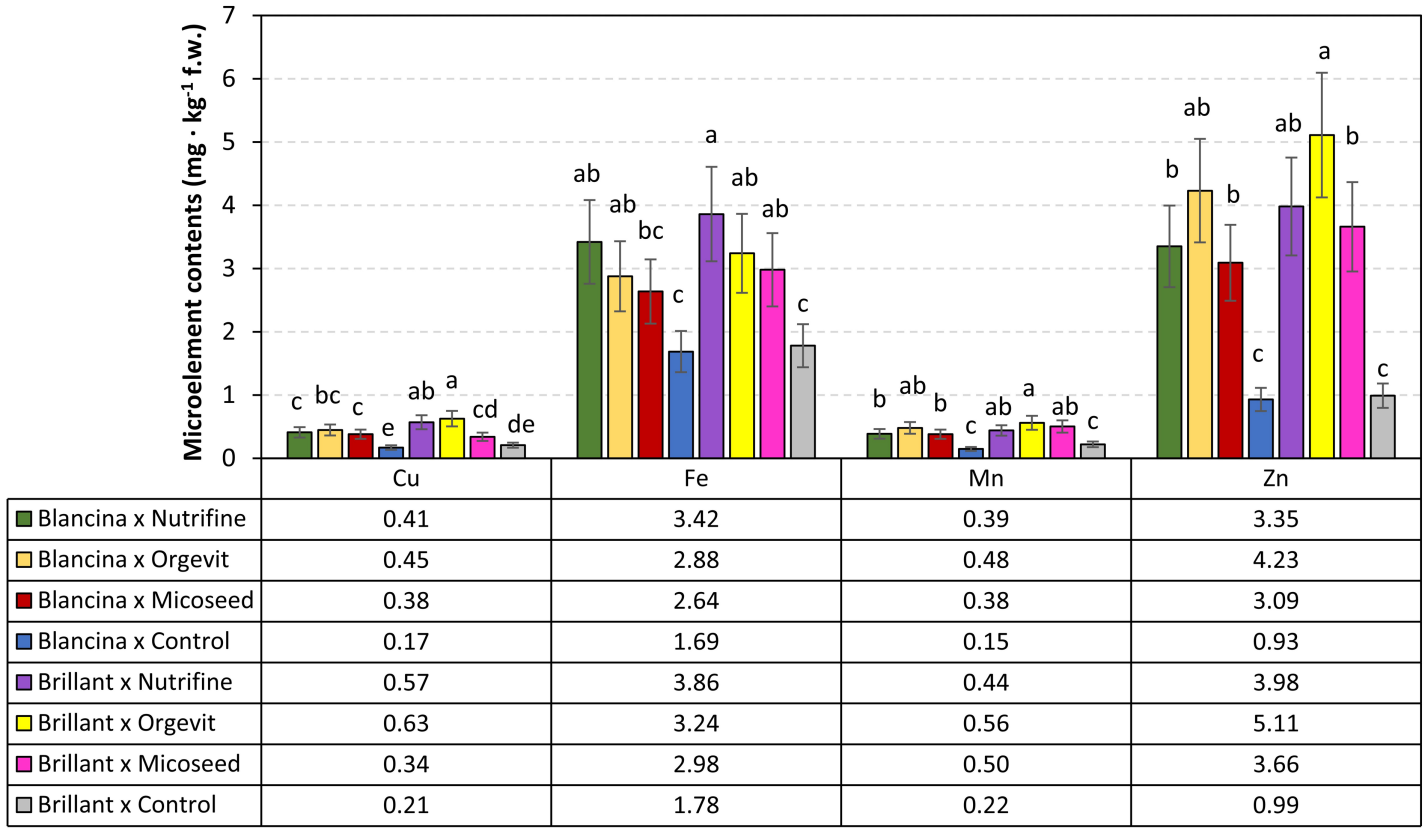
Total content of microelements in pepper fruits. Values associated with the same lower-case letters are not statistically different at *p* ≤ 0.05 according to Duncan’s test.

Regarding the microelements, fertilization of sweet peppers with Nutrifine^®^ resulted in the highest iron (Fe) content in their fruits, while the application of organic fertilizer led to the highest content of copper (Cu), manganese (Mn), and zinc (Zn) (Figure 2). However, a comparison of the means of each microelement content, related to each type of fertilization for the same cultivar., revealed that, overall, the differences were not statistically significant. The lowest levels of micro-elements were found in the Blancina’s fruits unfertilized (control). Statistical analysis of experimental data pointed out that the applied fertilizers induced significant differences in the microelement content of sweet peppers cultivars compared to the control. The use of Micoseed in the fertilization of peppers did not cause significant changes in the Cu content of Brillant fruits and Fe content of Blancina fruits.

## 4 Discussion

The present study aimed to investigate the effect of different type of fertilizers (chemical, organic, and biological) on the production, proximate analysis, phytochemical composition, and mineral content of two sweet pepper cultivars, Blancina and Brillant. The results obtained indicate that the type of fertilizer used can have a significant impact on the performance and nutritional quality of sweet peppers. Regarding the production indicators evaluated, the number of fruits increased significantly with chemical fertilization, whereas the weight of the fruits increased with all types of fertilization. In terms of yield per hectare, biological fertilization resulted in the highest yields, followed by chemical and organic fertilization. Barcanu et al. (37) investigated the effect of organic and chemical fertilization on two pepper cultivars, Regal and Cantemir, and demonstrated that the type of fertilization affects the Cantemir cultivar., while the Regal cultivar remains unaffected. These findings suggest that the choice of fertilizer should be based on the specific goals of the grower, as different fertilizers may lead to different results depending on the desired outcome. It is worth noting that although biological fertilization produced higher yields than chemical fertilization in the present study, this is not necessarily a universal rule. For example, Tahmasebi et al. (38) demonstrated that the chlorophyll content increased in organically fertilized peppers compared to chemically fertilized ones. However, the production did not increase, and both the number of flowers and fruits, as well as plant height, were not influenced by the type of fertilization. The effects of different type of fertilizers on crop performance may vary depending on a range of factors, including soil type, climate conditions, crop species, and management practices. Biological and organic fertilization contributes to the increase in soil enzymatic activity, which in turn enhances soil fertility by promoting its biological activity and the capacity for nutrient transformation and transport (39). Furthermore, fertilizers based on microorganisms, such as those used in this study (*Claroideoglomus etunicatum*, *Funneliformis mosseae*, *Glomus aggregatum*, *Rhizophagus intraradices*, *Trichoderma*, *Streptomyces*, *Bacillus*, and *Pseudomonas*), can aid in the solubilization of phosphorus in the soil, the second most important nutrient for plants after nitrogen, alongside nitrogen fixation and mineral absorption stimulation, thereby stimulating plant growth and ultimately increasing production. On the other hand, chemical fertilizers are rich in macronutrients N, P, K (40–42). Therefore, it is important to consider these factors when selecting a fertilizer for a specific crop and environment.

In terms of proximate composition, the results showed that biological fertilization had a positive effect on the dry matter, total soluble solids (TSS), and ash of the sweet peppers fruits, while chemical fertilization had a negative effect on these parameters. Barcanu et al. (37) obtained a higher content of TSS in the chemically fertilized peppers compared to the organically fertilized ones, for both Cantemir and Regal cultivars. However, the dry matter content was higher in the control group without any fertilization. TSS is an important quality indicator that includes soluble carbohydrates, organic acids, amino acids, and minerals. It can be stimulated by organic fertilization, as demonstrated by Brezeanu et al. (43), who achieved values of up to 7.61 Brix in *Capsicum annuum* L. var. *grossum* treated with biological foliar fertilization, compared to the control group with 6.59. In our study, the highest TSS values were obtained in both Blancina and Brillant cultivars with biological fertilization, reaching 6.32 and 6.83, respectively. Thus, we can observe a variation in quality influenced by both fertilization and genotype. In terms of consumer preferences, fruits with higher TSS content and color intensity will be more attractive (44). In contrast, all types of fertilization led to an increase in acidity, although the effect was more pronounced in response to biological fertilization in the case of the Brillant. Muscolo et al. (23) demonstrated that fertilization with vegetable compost waste resulted in the highest increase in the content of ascorbic acid and other compounds such as carbohydrates, vitamin E, polyphenols, carotenoids, and flavonoids in sweet peppers of the red Topepo cultivar. This effect was superior to the compost made from olive waste and horse dung. The superior performance of vegetable compost can be attributed to its richer content of macronutrients, which stimulate plant metabolism and the synthesis of antioxidant compounds. These results suggest that organic and biological fertilization may enhance the nutritional quality of sweet peppers, whereas chemical fertilization may have a detrimental effect on some of their attributes.

In this study, it was also found that all types of fertilization had a significant positive impact on the phytochemical composition and antioxidant activity of sweet peppers. Biological and organic fertilization resulted in the most substantial improvements in the nutritional quality of the peppers, while chemical fertilization had a less pronounced effect. Specifically, biological fertilization led to the highest total phenolic content of Blancina and Brillant peppers, resulting in increases of 76 and 14% compared to the control, respectively. Furthermore, the analysis of individual phenolic compounds in this study, such as chlorogenic acid, p-coumaric acid, quercetin, and isoquercetin, revealed an increased concentration of these compounds with biological fertilization, particularly in the Brillant cultivar compared to Blancina. Polyphenols are bioactive compounds produced through secondary metabolism and play essential physiological roles such as pigmentation, growth, reproduction, as well as plant protection against ultraviolet radiation, heat, herbivores, and pathogens. They are widely recognized as beneficial phytochemicals for human health (45). The synthesis of these compounds can be influenced by both abiotic factors such as light and biological factors like fertilization with microorganisms (46). Fertilization with microorganisms has been shown to increase the polyphenol content not only in peppers but also in other species like tomato or basil (47). For instance, Apostol et al. (48) demonstrated that fertilization with *Trichoderma atroviride* in six cultivars and a hybrid of long pepper (*Capsicum annuum* L. var. *longum*) stimulated the synthesis of total polyphenols, ranging from 60 to 129 mEq GAE/g fresh sample in Doljan and Kaprima F1 varieties. This stimulation also enhanced the acidity and antioxidant activity. A high content of polyphenols in plants can be associated with the presence of stress factors. An increased concentration of phenols at the root level serves as chemo-attractants for *Rhizobia* and fungi, promoting symbiosis formation and binding to soil matter that will be metabolized by the soil’s bacterial flora. Consequently, this leads to increased soil porosity and bioavailability of essential nutrients such as copper, iron, boron, zinc, manganese, molybdenum, potassium, and magnesium (49).

In this study, organic fertilization resulted in the highest lycopene and β-carotene content, with increases of 77% and 72% for Blancina and 59% and 54% for Brillant, respectively. The antioxidant activity of peppers was also affected by fertilization, with the highest values observed for biological fertilization. Peppers are an important source of these compounds for human nutrition, renowned for their antioxidant properties. The content of these carotenoids can vary depending on the species, cultivar., fruit ripeness, and cultivation conditions. Generally, the carotenoid content in peppers increases with fruit ripening and decreases during processing (50, 51). The positive effects observed in this study with the use of organic fertilizer based on poultry manure can be attributed to the high bioavailability of macronutrients. Organic NPK fertilizers enhance soil fertility, improve the physical properties of the soil, and serve as a substrate for soil microorganisms. As a result, microbial activity is increased, leading to the decomposition of organic material and the release of nutrients that can be readily taken up by plants. Ultimately, these factors contribute to an increase in production (15). Finally, the study found that different treatments had a significant impact on the mineral content of peppers, with all treatments resulting in increased levels of minerals such as potassium, calcium, and phosphorus. Notably, the chemical treatment led to the highest levels of these minerals. These results suggest that the choice of fertilizer may also affect the mineral content of sweet peppers. For example, Guilherme et al. (52) demonstrated that the nutrient content in Entinas pepper variety showed higher levels of Ca, Cu, K, and P with organic fertilization using horse manure, regardless of the states of ripeness, compared to chemical fertilization. Moreover, Rubio et al. (53) emphasized that in organic systems, the mineral values for Ca, Fe, Cu, and Zn are higher compared to conventional farming. However, for K, Mg, and Mn, the values tend to be lower in organic farming. The present study highlights the importance of selecting the appropriate fertilizer to optimize the production and nutritional quality of sweet peppers. The findings suggest that biological and organic fertilizers may provide more significant benefits in terms of nutritional quality than chemical fertilizers, although the specific effects may depend on the crop species and growing conditions. Therefore, growers should consider the specific needs of their crops and environment when selecting a fertilizer. Additionally, according to the results of this study, that different fertilization treatments may be used to enhance the mineral content of sweet peppers, which could have important implications for their nutritional value and health benefits.

## 5 Conclusion

The present study indicates that the type of fertilizer used has a significant impact on the production, proximate analysis, phytochemical composition, and mineral content of sweet peppers. Organic and biological fertilizers had a more pronounced effect on the nutritional quality of the sweet peppers than chemical fertilizers. Moreover, according to the results in this study, different treatments can be used to enhance the mineral content of sweet peppers, which could have essential implications for the nutritional value of these crops. Overall, the use of organic and biological fertilizers is recommended to improve the nutritional value of sweet peppers, which could benefit both the consumer and the producer. However, several limitations and future perspectives arise, taking into account the results of this study. In terms of limitations, it is difficult to appreciate if the increases in the nutritional value of the cultivars used in this research due to organic and biological fertilizers are sufficient to have a positive impact on health. Also, it cannot be said how much and how often sweet peppers should be consumed to be beneficial for health. In addition, the lack of data regarding the antinutritional composition of sweet peppers (phytic acid, tannins, oxalates, saponins, α-amylase, and trypsin) can limit our understanding about the bioavailability of minerals such as zinc, calcium or iron in human nutrition. Finally, the results are valid only for the cultivars tested and the environmental conditions present in this study. In the case of other factors, the results might differ.

Regarding the perspectives, further research is necessary in order to obtain high yields and superior qualities of sweet pepper. For instance, it should be investigated the effect of combined fertilizers (chemical, organic, biologic), in different dosages and at different peppers’ growth stages on the yield and qualities of sweet peppers. Also, long-term effect of the different types of fertilization on the growth, yield and nutritional quality of sweet peppers should be taken into consideration.

## Data availability statement

The raw data supporting the conclusions of this article will be made available by the authors, without undue reservation.

## Author contributions

VS: Conceptualization, Methodology, Writing – original draft. IM: Data curation, Formal analysis, Investigation, Writing – original draft. DA-M: Conceptualization, Formal analysis, Writing – original draft. GC-T: Formal analysis, Investigation, Methodology, Writing – original draft. AC: Investigation, Methodology, Software, Writing – original draft. OR-R: Supervision, Visualization, Writing – original draft, Writing – review & editing. MB: Formal analysis, Investigation, Writing – original draft. GM: Methodology, Software, Writing – review & editing. MR: Investigation, Methodology, Visualization, Writing – original draft. GC: Supervision, Validation, Writing – review & editing. AS: Methodology, Supervision, Validation, Visualization, Writing – original draft. GJ: Funding acquisition, Methodology, Resources, Supervision, Writing – original draft.
